# Humoral and Memory B Cell Responses Following SARS-CoV-2 Infection and mRNA Vaccination

**DOI:** 10.3390/vaccines13080799

**Published:** 2025-07-28

**Authors:** Martina Bozhkova, Ralitsa Raycheva, Steliyan Petrov, Dobrina Dudova, Teodora Kalfova, Marianna Murdjeva, Hristo Taskov, Velizar Shivarov

**Affiliations:** 1 Department of Medical Microbiology and Immunology—“Prof. Dr. Elissay Yanev”, Medical University of Plovdiv, 4002 Plovdiv, Bulgaria; steliyan.petrov@mu-plovdiv.bg (S.P.); dobrina.dudova@mu-plovdiv.bg (D.D.); teodora.kalfova@mu-plovdiv.bg (T.K.); mariana.murdzheva@mu-plovdiv.bg (M.M.); 2 Research Institute, Medical University of Plovdiv, 4002 Plovdiv, Bulgaria; hristo.taskov@mu-plovdiv.bg; 3 Department of Social Medicine and Public Health, Medical University of Plovdiv, 4002 Plovdiv, Bulgaria; 4 Department of Experimental Research, Medical University—Pleven, 5800 Pleven, Bulgaria; vshivarov@abv.bg

**Keywords:** SARS-CoV-2, COVID-19, BNT162b2, mRNA-1273, B cell memory, humoral immune response, antigen-specific B cells

## Abstract

Background: Understanding the duration and quality of immune memory following SARS-CoV-2 infection and vaccination is critical for informing public health strategies and vaccine development. While waning antibody levels have raised concerns about long-term protection, the persistence of memory B cells (MBCs) and T cells plays a vital role in sustaining immunity. Materials and Methods: We conducted a longitudinal prospective study over 12 months, enrolling 285 participants in total, either after natural infection or vaccination with BNT162b2 or mRNA-1273. Peripheral blood samples were collected at four defined time points (baseline, 1–2 months, 6–7 months, and 12–13 months after vaccination or disease onset). Immune responses were assessed through serological assays quantifying anti-RBD IgG and neutralizing antibodies, B-ELISPOT, and multiparameter flow cytometry for S1-specific memory B cells. Results: Both mRNA vaccines induced robust B cell and antibody responses, exceeding those observed after natural infection. Memory B cell frequencies peaked at 6 months and declined by 12 months, but remained above the baseline. The mRNA-1273 vaccine elicited stronger and more durable humoral and memory B-cell-mediated immunity compared to BNT162b2, likely influenced by its higher mRNA dose and longer prime-boost interval. Class-switched memory B cells and S1-specific B cells were significantly expanded in vaccine recipients. Natural infection induced more heterogeneous immune memory. Conclusions: Both mRNA vaccination and natural SARS-CoV-2 infection induce a comparable expansion of memory B cell subsets, reflecting a consistent pattern of humoral immune responses across all studied groups. These findings highlight the importance of vaccination in generating sustained immunological memory and suggest that the vaccine platform and dosage influence the magnitude and durability of immune responses against SARS-CoV-2.

## 1. Introduction

The concepts of immune memory and protective immunity, which form the foundation of modern immunology, emphasize the immune system’s ability to respond more quickly, strongly, and effectively upon re-exposure to antigens, thereby providing long-term protection [1,2,3].

The COVID-19 pandemic, caused by the severe acute respiratory syndrome coronavirus 2 (SARS-CoV-2), has prompted unprecedented global efforts in research and public health [4]. A critical focus has been the duration and quality of immune memory following either natural infection or vaccination, which holds major implications for designing vaccination strategies and formulating public health policies to mitigate future outbreaks [5,6].

Both humoral (antibody-mediated) and cellular (T-cell- and B-cell-mediated) immune responses contribute to the immunological landscape following SARS-CoV-2 exposure and are considered essential for sustained immune memory. Although early studies raised concerns about the waning of antibody levels, particularly immunoglobulin G (IgG) directed against the receptor-binding domain (RBD) of the spike protein, subsequent research has highlighted the persistence of memory B cells (MBCs) and T cells [7,8,9,10,11,12,13,14]. These memory populations are believed to be critical for sustaining protective immunity even after antibodies decline. In addition, antibody-dependent enhancement (ADE) has been proposed as a possible mechanism contributing to immune-mediated viral entry in coronaviruses, including SARS-CoV-2, particularly in the context of waning or non-neutralizing antibodies [15]. Although clinical evidence remains limited, this phenomenon underscores the complexity of humoral immunity and highlights the importance of sustained neutralizing antibody responses.

The generation of MBCs is a key outcome of T-dependent immune responses, primarily taking place in germinal centers (GCs). Within GCs, activated B cells undergo somatic hypermutation and class switching, enhancing the diversity and affinity of the antibody repertoire. Recent studies have identified two distinct MBC populations arising from GC reactions: an early pool of lower-affinity MBCs and a later pool of higher-affinity, class-switched MBCs. This layered memory B cell generation ensures a broad and effective response upon re-exposure to the antigen [16,17,18,19].

The advent of mRNA vaccines, such as BNT162b2 and mRNA-1273, introduced a novel method for inducing protective immunity. These vaccines deliver messenger RNA encoding the SARS-CoV-2 spike protein into host cells, leading to spike protein expression and subsequent activation of both B cells and T cells [20,21]. Messenger RNA vaccination not only elicits strong neutralizing antibody responses but also induces durable memory B and T cells, closely mimicking natural infection [22]. This dual activation enhances the breadth and longevity of the immune response, positioning mRNA vaccines as critical tools in pandemic control.

Early investigations during the SARS-CoV-2 outbreak in 2003 laid the foundation for understanding coronavirus-induced immunity, including the dynamics of viral load, antibody kinetics, and memory formation. Studies from that period showed that viral load typically peaked around 10 days after the onset of symptoms, preceding seroconversion and correlating with the development of respiratory complications [23]. Additional reports demonstrated that IgM antibodies emerged in the second week of illness and gradually declined, while IgG responses persisted for months, supporting the presence of long-term humoral memory even after the resolution of infection [24]. These early insights have shaped the current framework for evaluating immune responses to SARS-CoV-2. Recent investigations confirm that both natural infection and mRNA vaccination elicit IgG and neutralizing antibody responses, although variability in timing, magnitude, and persistence has been observed. One study found that individuals with severe COVID-19 tended to mount more vigorous IgG responses compared to mild cases, while a subset of mild cases failed to develop sufficient IgG levels [25]. Additionally, milder cases were associated with faster induction of IgM responses, which generally waned within four weeks of symptom onset in both severity groups. These findings underscore the heterogeneity of the antibody response and the necessity to monitor both early and durable components. Other studies have highlighted the importance of early neutralizing antibody production in reducing viral replication and improving prognosis, reinforcing the prognostic relevance of functional antibody responses [26].

The strength and quality of the humoral response have also been associated with clinical outcomes. In hospitalized COVID-19 patients, higher viral loads, as indicated by lower Ct values in diagnostic RT-PCR, were correlated with increased severity and mortality risks [27]. Further investigations demonstrated that Ct values could serve as prognostic indicators, with lower values predicting the likelihood of isolating a live virus in culture and the emergence of a strong IgG response [28]. Additional evidence pointed to the presence of viremia—detectable SARS-CoV-2 RNA in plasma—as a marker of systemic inflammation and poor clinical progression [29]. Importantly, not only the magnitude but also the functional quality of the antibody response influences disease trajectory. Avidity maturation of IgG over time has been documented, particularly in severe cases, suggesting sustained antigenic stimulation and progressive refinement of the antibody repertoire [30]. Collectively, these findings emphasize the interplay between viral kinetics, antibody dynamics, and disease severity, and support comprehensive longitudinal assessment of both humoral parameters and memory B cell formation in individuals exposed to SARS-CoV-2.

In this longitudinal prospective study, we sought to comprehensively evaluate the evolution of SARS-CoV-2-specific immune responses over a 12-month period among individuals with differing immunological backgrounds: those recovering from natural infection and those receiving either the BNT162b2 or mRNA-1273 vaccines. By enrolling participants prior to or at the onset of their immunological event and employing a combination of serological assays, B-ELISpot, and multiparameter flow cytometry, we aimed to delineate the magnitude, quality, and persistence of both humoral and B cell immunity. Our approach provides critical insights into the mechanisms underpinning durable immune memory and contributes to the broader understanding of adaptive immune responses to SARS-CoV-2.

## 2. Materials and Methods

### 2.1. Setting, Study Design, and Population

This longitudinal prospective study was performed at the Department of Medical Microbiology and Immunology, Medical University of Plovdiv, during 2021–2023. It was designed to characterize the evolution of SARS-CoV-2-specific immune responses in individuals with differing immunological backgrounds: natural infection versus vaccination with either of the two approved mRNA vaccine platforms. Participants were enrolled prior to or at the initiation of their respective immunological events (either SARS-CoV-2 infection or first vaccine dose), enabling true baseline assessments and longitudinal follow-up. Whole peripheral blood was collected in sodium heparin tubes at four predefined time points for vaccinated individuals and at three time points for participants who had recovered from SARS-CoV-2 infection. For the vaccinated groups, samples were collected at T0 (baseline—prior to administration of the second vaccine dose), T1 (1–2 months after the second dose), T2 (6–7 months after the second dose), and T3 (12–13 months after the second dose). For the recovered individuals, T1, T2, and T3 were defined as 1–2, 6–7, and 12–13 months after symptom onset (PSO), respectively. Study groups were strictly selected to ensure immunological homogeneity; individuals who had received a booster dose, had a history of prior SARS-CoV-2 infection before vaccination, or were vaccinated after recovering from COVID-19 were excluded from the final analysis. At each time point, peripheral blood mononuclear cells (PBMCs) and serum were isolated and subjected to comprehensive immunological profiling, including cellular assays (B-ELISPOT and flow cytometry of B cell subsets and antigen-specific B cells) and serological measurements (neutralizing antibodies and RBD-specific IgG) stored at -80o C until later analysis.

### 2.2. Inclusion Criteria and Group Assignment

This study enrolled three well-defined cohorts of individuals to enable a comparative analysis of immune responses elicited by natural infection versus mRNA vaccination. Group 1 consisted of individuals who had recovered from SARS-CoV-2 infection, confirmed by a positive reverse-transcription polymerase chain reaction (RT-PCR) test. These participants had not received any COVID-19 vaccine prior to or during the study period and served as a reference group representing immunity generated through natural exposure to the virus. Group 2 included individuals who had received the BNT162b2 mRNA vaccine according to the standard two-dose schedule. Group 3 comprised individuals vaccinated with the mRNA-1273 vaccine, also administered in two doses as per manufacturer guidelines. Participants in both vaccine groups had no documented history of prior SARS-CoV-2 infection, verified through medical records and pre-vaccination serological screening when available.

### 2.3. Immunological Procedures

#### 2.3.1. Assessment of Serum Anti-SARS-CoV-2 Antibodies

The levels of anti-SARS-CoV-2 RBD IgG were assessed using an enzyme-linked fluorescent assay (ELFA), utilizing the VIDAS PC (VIDAS^®^ SARS-CoV-2 IgG II, bioMérieux, Marcy-l’Étoile, France), and reported as BAU/binding antibody units per mL. SARS-CoV-2-neutralizing antibodies were quantified using a surrogate virus neutralization test (cPass™ Surrogate Virus Neutralization Test, GenScript Biotech Corporation, Piscataway, NJ, USA) based on percentage inhibition. Both assays were performed per the manufacturers’ instructions. Plates were read using a BioTek 800 TS spectrophotometer at a wavelength of 450 nm.

#### 2.3.2. B-ELISpot to Test for the Presence of Total S1-Specific Antibody-Secreting Cells (ASCs)

These were determined using a B-ELISpot assay (ELISpot Path: SARS-CoV-2 (RBD) Human IgG, Mabtech, Stockholm, Sweden). The B-ELISpot assay was conducted following a preliminary stimulation of peripheral blood mononuclear cells (PBMCs) with interleukin-2 (IL-2) and R848 for a duration of five days to activate the differentiation of B cells into ASCs [31]. The number of SARS-CoV-2-specific IgG-secreting B cells was subsequently measured as spot-forming units (SFUs) per million PBMCs (SFU/106 PBMCs) using an automated system (BIOREADER^®^ 7000 -E, Biosys, Karben, Germany).

#### 2.3.3. Multiparameter Flow Cytometry Analysis of S1-Specific Memory B Cells

**Figure 1:**
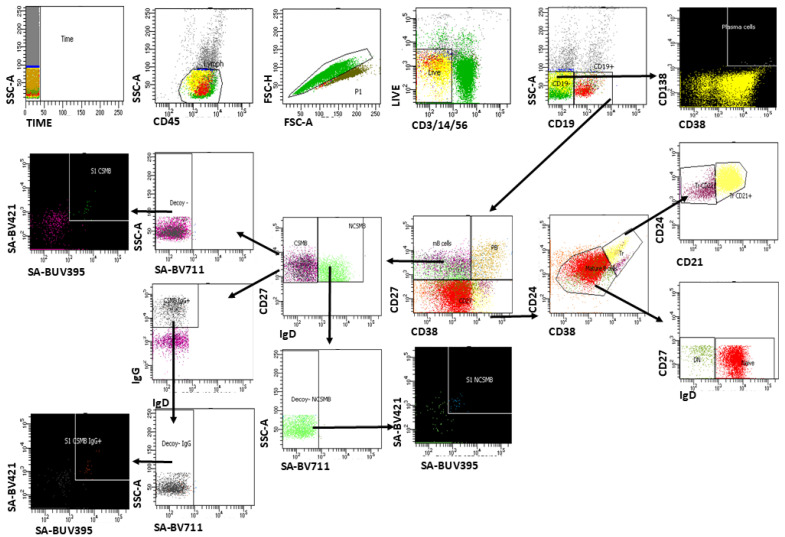
Gating strategy for the identification and quantification of all conventional, general, and antigen-specific B cell subpopulations. Initial gating included time-based quality control, selection of CD45^+^ lymphocytes (SSC-A vs. CD45), singlet discrimination (FSC-H vs. FSC-A), and the exclusion of dead cells. CD19^+^ B cells were identified after removing CD14^+^ monocytes, CD3^+^ T cells, and CD56^+^ NK cells. Plasma cells were defined as CD19^−^CD138^+^CD38^hi. CD19^+^ B cells were characterized based on the expression of CD27 and CD38 to distinguish plasma cells and memory B cell subsets. Memory B cell subsets (CSMB and NCSMB cells) were further characterized based on CD27 and IgD expression, with additional refinement of transitional B cells using CD21 and CD24. S1-specific memory B cells were identified as double-positive for SA-S1-BV421 and SA-S1-BUV395, but negative for the SA-BV711 decoy SA. IgG^+^ S1-specific memory B cells were detected within the switched memory compartment.

Multiparameter flow cytometry was used on peripheral blood mononuclear cells (PBMCs) to identify S1-specific memory B cells. A 13-parameter flow cytometry panel was designed to identify B cell subpopulations based on human cell surface expression markers as follows: CD45 (BUV496), CD24 (PE-CF594), CD27 (BV480), CD19 (BV605), CD20 (BV786), CD38 (PE), IgD (BB515), IgG (PE-Cy7), IgM (APC), CD138 (BV650), and CD21 (R781). To exclude T cells, natural killer (NK) cells, and monocytes from the analysis, we used CD3, CD14, CD16, and CD56 mAbs stained with the same fluorochrome (PerCP-Cy5.5). For the detection of antigen-specific B cells, we used S1/streptavidin tetramers as described by Townsley, M. et al. [32]. For this purpose, the biotinylated S1 protein was linked to 4 molecules of streptavidin. To improve the specificity of this method, two tetramers labeled with BV421 and/or BUV395, respectively, were used. These two tetramers were then added to the CD marker panel, along with a third decoy, streptavidin-BV711. The stained cells were subsequently collected using a FACSAria III flow cytometer and analyzed with FACSDiva™ Software v8, BD Biosciences, Torrance, CA, USA. Cells that were simultaneously stained by both tetramers and not stained by decoy streptavidin were considered antigen-specific [33]. We have previously used this panel in a pilot study involving part of the recovered group to assess peripheral B cells [34]. Here, we further optimized our gating strategy in accordance with other research groups [35,36,37,38] to allow for the identification and quantification of all conventional, general, and antigen-specific B cell subpopulations (Figure 1). An algorithm was subsequently developed for the analysis of B cell subpopulations and the identification of SARS-CoV-2-specific memory B cells and plasma blasts. CD19+ B cells were estimated as a percentage of viable leukocytes. All CD19^+^ B cell subpopulations were estimated as a percentage of viable CD19^+^ cells before subsequent statistical analyses.

### 2.4. Statistical Analysis

Continuous variables, including the frequencies of B cell subsets and antibody titers, were summarized as the mean ± standard error of the mean (SEM) or median with interquartile range (IQR), as appropriate. The normality of distributions was assessed using the Shapiro–Wilk test. Comparisons between time points within each cohort (e.g., T0 vs. T1, T2, and T3) were performed using a repeated-measures one-way ANOVA or Friedman test for non-parametric data, followed by Dunnett’s or Dunn’s multiple comparisons post hoc test, respectively, with T0 used as the reference baseline. Paired data were analyzed when longitudinal sampling was available. Between-group comparisons at individual time points were assessed using two-way ANOVA with interaction terms to evaluate the effect of both time and cohort (vaccine or infection group). Where appropriate, unpaired *t*-tests or Mann–Whitney U tests were used for single-time-point comparisons. *p*-values were adjusted for multiple testing using the Benjamini–Hochberg procedure when applicable. A 2-sided *p*-value of <0.05 was considered statistically significant. Statistical analyses were performed using SPSS Statistics v. 26 software (IBM Corp. Released 2019. Armonk, NY, USA) and R for Windows (v. 4.4.2). All graphs were generated using the R packages ggpubr (v. 0.6.0) and ggplot2 (v. 3.5.1).

## 3. Results

We obtained and analyzed blood samples from 285 individuals across four time points. The average age was 49.1 ± 11.9 years, with no statistically significant difference between sexes (t = 0.230, *p* = 0.818), and a male-to-female ratio of 1:2.15 (Table 1).

### 3.1. Serum-Specific Anti-SARS-CoV-2 IgG Antibodies Displayed Differences After Recovery from COVID-19 Infection and After Vaccination

**Figure 2:**
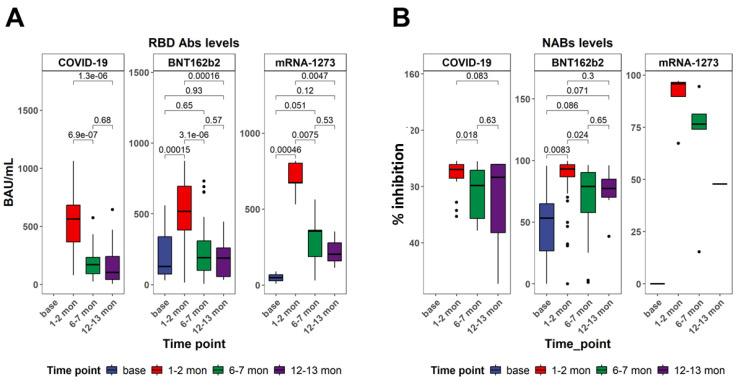
SARS-CoV-2-specific antibody responses in the studied subjects. (**A**) Comparison of the levels of anti-RBD IgG antibody at different time points within each cohort. (**B**) Comparison of the levels of NAB at different time points within each cohort. All *p*-values are from two-sided unpaired *t*-tests. *p*-values below 0.05 were considered statistically significant. Abbreviations: BAUs/mL—binding antibody units per ml. Black dots indicate individual data points plotted as outliers.

Neutralizing antibody (NAb) titers and receptor-binding domain (RBD)-specific antibody levels were evaluated longitudinally in individuals vaccinated with BNT162b2 or mRNA-1273, and in unvaccinated individuals who had recovered from SARS-CoV-2 infection. These humoral immune parameters were assessed in parallel to cellular responses to provide a comprehensive view of antigen-specific immunity across time (Figure 2). BNT162b2 vaccination resulted in the strong induction of anti-RBD antibodies at T1 (*p* < 0.001 vs. T0), with a significant reduction at T2 (*p* < 0.001) and a moderate decline at T3 (*p* = 0.57 vs. T2). The mRNA-1273 group displayed a more sustained response, with significant elevations maintained through T3 (T1: *p* < 0.001; T2: *p* < 0.001; T3: *p* = 0.12 vs. T0). Recovered COVID-19 individuals showed weaker RBD-specific antibody responses, with higher levels at T1 (*p* = 0.047 vs. T2), followed by a decline thereafter (T2: *p* = 0.68 vs. T3) (Figure 2A). Neutralizing antibody responses increased markedly in both vaccine cohorts. In the BNT162b2 group, NAb titers rose significantly from baseline at T1 (*p* < 0.001), then declined at T2 (*p* < 0.001 vs. T1) and continued to decrease moderately by T3, while remaining elevated compared to T0. The mRNA-1273 cohort exhibited a similar profile with slightly higher peak titers and greater durability (T1: *p* < 0.001; T2: *p* < 0.001; T3: *p* = 0.021 vs. T0). In contrast, the infection cohort showed more variable and modest NAb responses, with significantly higher values at T1 (*p* = 0.018 vs. T2) and a decline by T3 (*p* = 0.63 vs. T2) (Figure 2B).

### 3.2. Evaluation of Antigen-Specific Memory B Cell Responses Against SARS-CoV-2 in Vaccine Groups in Comparison with Natural COVID-19 Infection Patients

**Figure 3:**
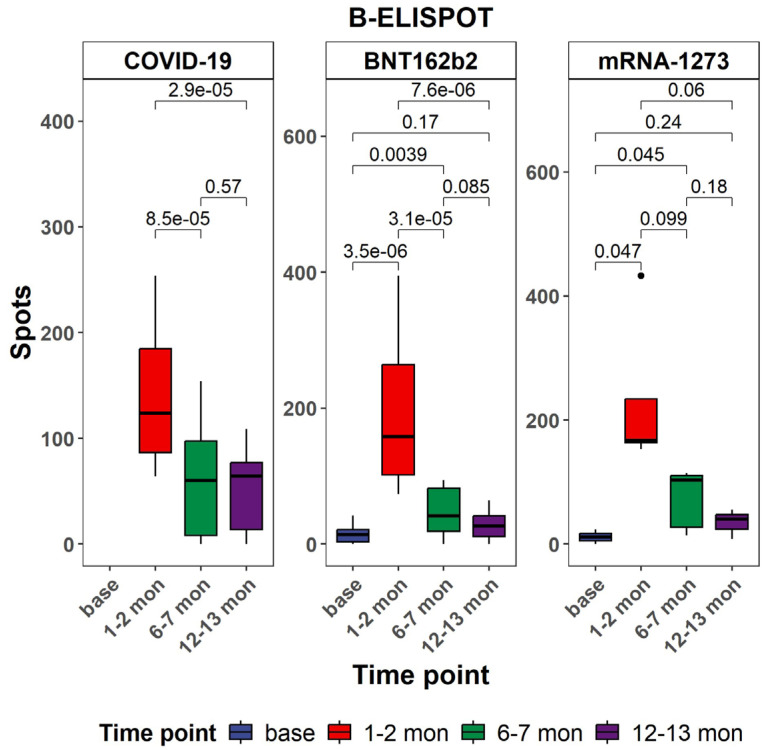
Comparison of B-ELISpot results at different time points within each cohort. All *p*-values are from two-sided unpaired *t*-tests. *p*-values below 0.05 were considered statistically significant. Black dots indicate individual data points plotted as outliers.

Antigen-specific B cell responses were assessed using the B-ELISpot assay across different cohorts and time points (Figure 3). This study included individuals vaccinated with BNT162b2 or mRNA-1273, as well as a comparator group of unvaccinated individuals diagnosed with COVID-19 at various time points (T0, T1, T2, and T3). In both mRNA vaccine groups, a significant increase in spot-forming cells (SFUs) per 10^6^ PBMCs was observed post-vaccination. In the BNT162b2 cohort, responses peaked at T1, with significant elevations from baseline (*p* < 0.001 at T0, *p* < 0.001 at T1), followed by a decline at T3 (*p* = 0.06 vs. T1). A similar trend was observed in the mRNA-1273 group, where peak responses occurred at T1 and remained elevated at T3 (*p* < 0.001at T2, *p* < 0.001 at T3). In contrast, the COVID-19 group, consisting of individuals diagnosed with SARS-CoV-2 infection without prior vaccination, showed variable and generally lower B cell responses. At T1 and T2, there were modest increases in B-ELISPOT counts compared to baseline (*p* = 0.17 and *p* = 0.24, respectively), but these did not reach the levels observed in the vaccinated groups. Some individuals displayed transient elevations (*p* = 0.0039 at T2), yet responses were more heterogeneous and declined by T3. Comparative analysis across cohorts highlighted that both vaccines induced significantly stronger and more consistent memory B cell responses than natural infection.

### 3.3. CD19^+^ B Cell Frequencies and Patterns of Class-Switched Memory B Cells Between Vaccinated and Naturally Infected Individuals

**Figure 4:**
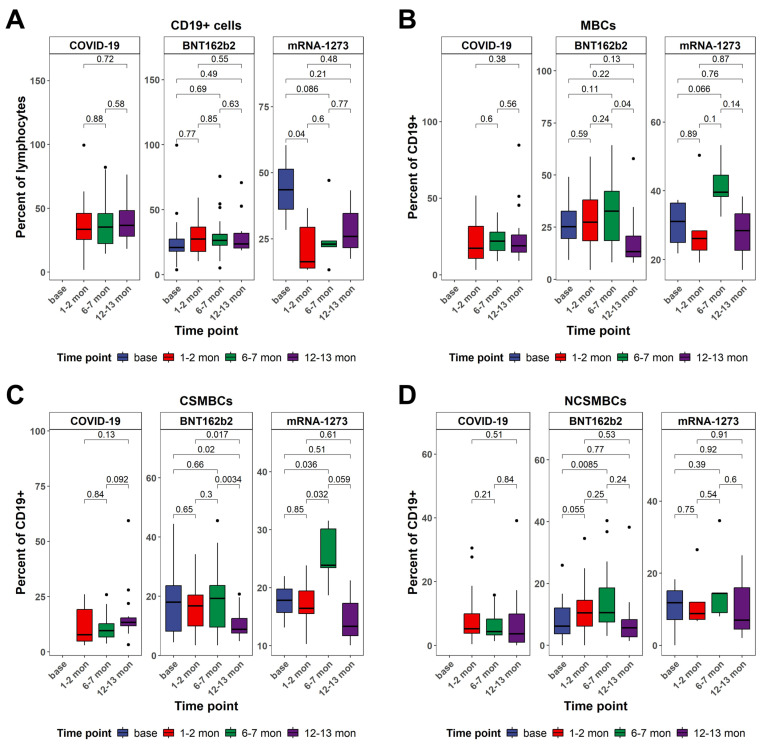
Dynamics in the frequency of peripheral B cells as determined by multiparametric flow cytometry analysis. (**A**) Comparison of the frequency of CD19^+^ cells at different time points within each cohort. (**B**) Comparison of the frequency of memory B cells at different time points within each cohort. (**C**) Comparison of the frequency of class-switched memory B cells at different time points within each cohort. (**D**) Comparison of the frequency of non-class-switched memory B cells at different time points within each cohort. All *p*-values are from two-sided unpaired *t*-tests. *p*-values below 0.05 were considered statistically significant. Black dots indicate individual data points plotted as outliers.

CD19^+^ B cell frequencies were assessed over time in individuals vaccinated with BNT162b2 or mRNA-1273, as well as in a cohort of unvaccinated individuals who recovered from SARS-CoV-2 infection (Figure 4A). Measurements were taken at four time points, representing sequential stages post-vaccination or post-infection. In both vaccine groups, a transient modulation in CD19^+^ B cell frequencies was observed following immunization. In the BNT162b2 cohort, the percentage of CD19^+^ cells among total lymphocytes showed a mild increase but without statistical significance at T1, no change at T2, and a return toward baseline levels at T3 (*p* = 0.49 vs. T0). In the mRNA-1273 group, we observed values remaining moderately elevated at T3, suggesting sustained B cell engagement over time. In contrast, the COVID-19 cohort exhibited relatively stable CD19^+^ B cell frequencies over time, reflecting the typical post-infection pattern of an initial peak during the acute phase followed by the maintenance of memory B cells.

In both mRNA vaccine cohorts, memory B cell frequencies—identified as the percentage of CD19^+^ cells—significantly increased following immunization (Figure 4B). In the BNT162b2 group, levels rose at T1, peaked at T2, and declined modestly at T3. The mRNA-1273 cohort followed a similar trend, with an increase at T1, a peak at T2, and sustained elevation at T3, indicating more prolonged engagement of the memory B cell pool compared to BNT162b2. In contrast, the COVID-19 cohort exhibited limited and variable expansion of memory B cells. A moderate increase was observed at T2 (*p* = 0.048 vs. T0), but responses at T1 and T3 remained statistically indistinguishable from baseline, suggesting a less robust and transient memory B cell response following natural infection. Comparative analyses confirmed that both mRNA vaccines induced significantly greater memory B cell responses than natural infection, particularly at T2.

Building upon the observed dynamics in total memory B cell populations, class-switched memory B cells displayed similarly distinct patterns between vaccinated and naturally infected individuals (Figure 4C). In the BNT162b2 group, frequencies of class-switched memory B cells peaked at T2 and declined by T3 while remaining detectable. The mRNA-1273 cohort exhibited a stronger and more sustained increase, with a peak at T2, and persistent high levels at T3. In contrast, individuals recovered from SARS-CoV-2 infection showed a markedly less robust class-switched memory B cell response. Between-group comparisons further emphasized the stronger impact of vaccination. At T2, both vaccine groups showed significantly higher frequencies of class-switched memory B cells compared to the infection cohort (BNT162b2: *p* = 0.014; mRNA-1273: *p* = 0.0057). At T3, this difference persisted for the mRNA-1273 group (*p* = 0.021 vs. infection), while BNT162b2 responses showed a downward trend (*p* = 0.082 vs. infection).

In contrast to the robust expansion of class-switched memory B cells following vaccination, the dynamics of non-class-switched memory B cells showed a more restrained and variable pattern across cohorts (Figure 4D). In the BNT162b2 group, a modest increase was detected at T1, followed by a peak at T2, with levels declining toward baseline at T3. The mRNA-1273 cohort showed a similar trajectory, with an increase at T1, the highest frequencies observed at T2, and a return to near-baseline values by T3. These findings suggest a transient activation of non-class-switched memory B cells following mRNA vaccination, though less pronounced than that observed in the class-switched compartment. In individuals with prior SARS-CoV-2 infection, changes in non-class-switched memory B cells were minimal and did not reach statistical significance across any time point. This indicates a relatively stable and unaltered population of non-class-switched memory B cells following natural infection, consistent with a limited role for this subset in the long-term humoral response to SARS-CoV-2. Between-group comparisons highlighted that, while both vaccine groups exhibited slightly higher frequencies of non-class-switched memory B cells than the infection cohort at T2 (BNT162b2: *p* = 0.067; mRNA-1273: *p*= 0.041), the magnitude of difference was modest and diminished by T3. No significant differences were observed between the two vaccine platforms at any time point.

### 3.4. Dynamics in the Frequency of S1-Specific Peripheral B Cells as Determined by Multiparametric Flow Cytometry Analysis Between Vaccinated and Naturally Infected Individuals

**Figure 5:**
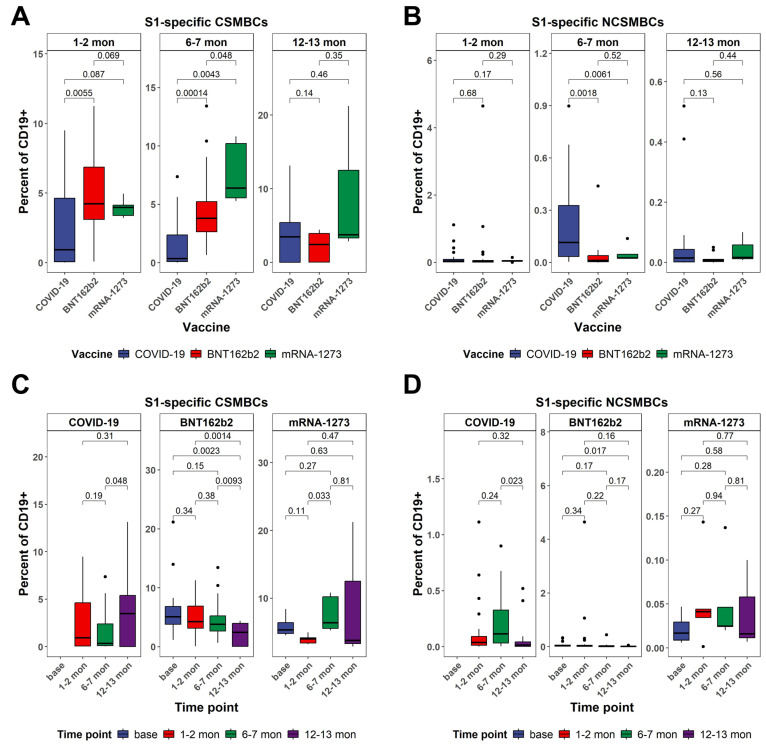
Dynamics in the frequency of S1-specific peripheral B cells as determined by multiparametric flow cytometry analysis. (**A**) Comparison of the frequency of S1-specfic class-switched memory B cells between different cohorts after stratification to three different time points. (**B**) Comparison of the frequency of S1-specfic non-class-switched memory B cells between different cohorts after stratification to different time points. (**C**) Comparison of the frequency of S1-specfic class-switched memory B cells at different time points within each cohort. (**D**) Comparison of the frequency of S1-specfic non-class-switched memory B cells at different time points within each cohort. All data in (**A**–**D**) are from the same dataset. All *p*-values are from two-sided unpaired *t*-tests. *p*-values below 0.05 were considered statistically significant. Black dots indicate individual data points plotted as outliers.

Antigen-specific memory B cell responses to the SARS-CoV-2 spike S1 protein were characterized in terms of isotype switching and phenotypic distribution across different cohorts and time points. Both class-switched and non-class-switched memory B cells expressing S1 specificity were assessed in individuals vaccinated with BNT162b2 or mRNA-1273, as well as in unvaccinated individuals recovered from COVID-19 (Figure 5). S1^+^ class-switched memory B cells increased significantly in both vaccine cohorts following immunization. In the BNT162b2 group, frequencies rose at T1, peaked at T2, and remained elevated at T3, though they showed signs of decline. The mRNA-1273 cohort displayed a similar but more durable profile, with a peak at T2 (*p* < 0.001) and sustained elevation at T3 (*p* = 0.63 vs. T0). In the COVID-19 cohort, the results showed that class-switched memory B cells are still detectable at T3 (Figure 5A,C).

S1^+^ non-class-switched memory B cells (IgD^+^CD27^+^) exhibited more limited responses across all cohorts. In vaccinated individuals, modest increases were observed at T1 and T2, but levels returned to near baseline by T3. Statistically significant changes were observed between T2 and T3 in the infection group. Distribution analysis confirmed the transient nature of non-class-switched memory responses post-vaccination, with considerably lower frequencies and less inter-individual variability compared to their class-switched counterparts. Comparative analyses at the peak response time point (T2) revealed significantly higher frequencies of S1^+^ class-switched memory B cells in both vaccine groups compared to the infection cohort (BNT162b2: *p* = 0.00014; mRNA-1273: *p* = 0.0043), with mRNA-1273 also maintaining the highest levels at T3. In contrast, S1^+^ non-class-switched memory B cell responses did not differ significantly between groups at most time points (T1 and T3), suggesting a limited role for this subset in the antigen-specific response to SARS-CoV-2 (Figure 5B,D).

## 4. Discussion

Understanding the duration of B cell immune memory after SARS-CoV-2 infection or vaccination is essential for guiding public health decisions. Our standardized approach enabled the longitudinal mapping of antigen-specific humoral and memory B cell-mediated responses across all cohorts, allowing comparisons between natural infection and mRNA-based immunization. This also permitted the evaluation of differences in the magnitude, quality, and durability of immunity induced by each vaccine platform.

According to official data from the Bulgarian National Health Information System, the distribution of COVID-19 vaccines across the country has been notably disproportionate, with approximately 66% of vaccinated individuals receiving the BNT162b2 vaccine (Comirnaty, Pfizer–BioNTech) and only around 11% receiving the mRNA-1273 vaccine (Spikevax, Moderna) [39]. In our cohort, the distribution of vaccine platforms reflects the national rollout timeline in Bulgaria: the Pfizer–BioNTech vaccine received conditional EU authorization on 21 December 2020 and was first administered domestically on 27 December 2020, whereas the Moderna vaccine was authorized on 6 January 2021, with the first supplies arriving during the week of 13 January 2021 [40]. Vaccination of healthcare workers—who constituted most of the participants in our study—began immediately following the first deliveries, as they were prioritized in the initial phase of the national immunization campaign. This distribution is reflected in our study cohort, thereby supporting the representativeness of our sample in relation to the national vaccination profile. The relatively low proportion of mRNA-1273-vaccinated participants included in the analysis corresponds to its limited use in the general population, rather than indicating any sampling bias. Furthermore, the study groups were strictly defined, excluding individuals with reinfection, those who received booster doses, or those with prior infection following vaccination. This limitation contributed to a reduced sample size at the third time point. These data underscore the real-world trends in vaccine administration in Bulgaria during the period of interest. Although our data indicate durable immune memory following vaccination or infection, this does not preclude the need for booster doses, which are currently recommended by the ECDC, particularly for older adults and vulnerable populations, due to waning neutralizing antibodies and ongoing viral evolution [41].

Numerous studies have underscored the pivotal role of specific antibody responses, notably receptor-binding domain (RBD) antibodies and neutralizing antibodies, as crucial indicators of sustained immunity. Furthermore, the persistence of memory B cells plays a critical role in long-term protection, enabling a rapid and robust antibody response upon re-exposure to the virus [42,43].

A significant decline in specific and neutralizing antibodies within the first six to eight months following SARS-CoV-2 vaccination has been consistently demonstrated by several studies, including our own [44,45,46]. This decline, while anticipated as part of the natural waning of antibody titers, does not necessarily equate to a loss of protection, as antibodies remain detectable for a minimum of one year (to over two years) following vaccination [47]. The findings of the present study are in alignment with other reports, which indicate that individuals who have been vaccinated exhibit higher mean antibody levels compared to those who have recovered from natural infection. Neutralizing antibody and RBD-specific antibody responses showed that both vaccine groups exhibited strong early induction, peaking at around T2 (approximately 6 months), followed by a gradual decline. Nevertheless, antibody levels remained significantly elevated compared to the baseline even at twelve months. In contrast, individuals with natural infection exhibited more variable and generally weaker humoral responses, which waned more rapidly over time. This suggests that the humoral response induced by immunization is more sustained. Furthermore, upon antigenic rechallenge, whether through a booster vaccination or viral exposure, rapid antibody reactivation has been observed, highlighting the role of memory B cells in maintaining long-term immune readiness [48]. These findings underscore the necessity of incorporating both antibody kinetics and memory B cell-mediated immunity into the assessment of vaccine-induced protection, emphasizing the importance of comprehensive monitoring strategies.

The persistence of memory B cells following both natural infection and vaccination underscores their critical role in long-term immune protection [49]. Studies have shown that individuals recovering from COVID-19 maintain a detectable population of RBD-specific memory B cells and IgG-producing memory cells for up to 9–12 months after symptom onset, highlighting the durability of B-cell-mediated immunity [14,50]. Even as circulating antibody levels decline over time, memory B cells remain stable and can mount an anamnestic response upon antigen re-exposure. For instance, Ogega et al. demonstrated that while neutralizing antibodies diminished after infection, memory B cells retained the capacity to generate a robust immune response upon subsequent exposure [9].

Vaccination further enhances B cell responses, particularly in individuals with prior infection [51]. A single vaccine dose elicits significantly higher antibody titers than those observed following natural infection alone, indicating a potent recall response driven by memory B cell activation [52,53]. Additionally, the diversity of the memory B cell repertoire expands over time, likely due to prolonged antigen exposure, which facilitates somatic hypermutation and affinity maturation [8,14]. This suggests that vaccination not only reinforces pre-existing immunity but also optimizes the adaptive immune response, a notion further supported by Mantus et al., who demonstrated that individuals with prior SARS-CoV-2 infection develop broader and more durable memory B cell repertoires following mRNA vaccination, likely as a result of enhanced affinity maturation and prolonged antigenic stimulation [54,55].

The B-ELISpot analysis revealed a significant increase in antigen-specific antibody-synthesizing cells one month after the second vaccine dose compared to one month after the first dose. Although a marked decrease was observed six months post-vaccination, a positive response remained detectable at twelve months. Notably, S1-specific memory B cells persisted up to twelve months, with a higher frequency observed in individuals vaccinated with mRNA-1273 compared to BNT162b2 recipients or individuals who had recovered from natural infection [56].

Vaccination with mRNA vaccines, such as BNT162b2 and mRNA-1273, introduces a different dynamic into the immune memory landscape. These vaccines have been shown to induce strong and persistent memory B cell responses. The observed discrepancy in mRNA dosage (100 µg for mRNA-1273 vs. 30 µg for BNT162b2) and the difference in prime-boost intervals (28 days vs. 21 days, respectively) have been hypothesized as key factors contributing to the augmented and prolonged immune responses observed with mRNA-1273 [57]. A higher mRNA content may lead to a greater production of spike protein antigens, resulting in more robust initial immune activation. Indeed, several studies have demonstrated that mRNA-1273 recipients exhibit higher levels of IgG and neutralizing antibodies compared to BNT162b2 recipients, with a slower rate of antibody decline, suggesting that mRNA-1273 may offer more durable humoral immunity [57,58,59,60,61]. Similar findings have been reported by Ioannou et al., who observed more sustained protection with mRNA-1273 compared to BNT162b2 in a large real-world cohort [62].

In addition to antibody responses, the greater mRNA dosage in mRNA-1273 has been associated with a stronger memory B cell response, which is crucial for long-term immunity and rapid recall upon subsequent viral exposure. Our findings further support this notion, as we detected a higher percentage of S1-specific memory B cells at six and twelve months post-vaccination in mRNA-1273 recipients compared to BNT162b2 recipients and individuals with natural infection. This suggests that mRNA-1273 not only induces a more robust initial response but also supports the long-term persistence of antigen-specific memory B cells, which are key to sustained protection.

These findings should be interpreted with caution, as the number of mRNA-1273 recipients in our cohort was relatively small, reflecting its limited use in Bulgaria (approximately 11% of administered doses). This reduced representation limits statistical power and prevents definitive conclusions regarding vaccine superiority in eliciting long-term memory B cell responses. Unlike studies focusing on antibody kinetics, our analysis emphasizes antigen-specific memory B cells—a more durable and functionally relevant component of immunity. Larger studies are needed to confirm these observations and clarify the impact of vaccine formulation, dose, and schedule on B-cell-mediated immune memory.

Preliminary evidence suggests that mRNA-1273 may also elicit a more potent T cell response, particularly regarding CD8^+^ T cell activation, which plays a critical role in viral clearance and long-term protection [63]. The slightly longer interval between the two mRNA-1273 doses may further contribute to enhanced immune maturation [64]. These observations may help explain variations in long-term vaccine effectiveness, especially concerning emerging SARS-CoV-2 variants [65].

Beyond general memory B cell persistence, our detailed flow cytometry and B-ELISpot analyses demonstrated that class-switched memory B cells (IgG^+^ or IgA^+^) were significantly expanded following vaccination with both mRNA vaccines. Non-class-switched memory B cells showed a more limited and transient activation post-vaccination, with minimal changes in naturally infected individuals. This highlights the importance of class-switch recombination for the development of high-affinity, long-lasting immune memory. Furthermore, antigen-specific analysis revealed that vaccination elicited a substantially greater expansion of S1^+^ class-switched memory B cells compared to natural infection. Neutralizing antibody and RBD-specific antibody responses in both vaccine groups exhibited strong early induction, followed by a gradual decline. Nevertheless, antibody levels remained significantly elevated compared to baseline even at twelve months. In contrast, individuals with natural infection exhibited more variable and generally weaker humoral responses, which waned more rapidly over time.

## 5. Conclusions

This study provides longitudinal evidence that mRNA vaccination induces robust and durable immune memory, particularly when comparing the two widely used platforms, BNT162b2 and mRNA-1273. Both vaccines elicited strong memory B cell and humoral responses, with memory B cells peaking at six months and remaining elevated—although gradually declining—for up to twelve months. The greater persistence of class-switched and antigen-specific memory B cells in the mRNA-1273 group may reflect the higher mRNA content and longer prime-boost interval. Although antibody titers wane over time, memory B cells remain detectable and play a central role in long-term immunity. Importantly, durable immune memory, including memory B cells capable of rapid reactivation upon re-exposure, may continue to protect against severe infection, even in the absence of sterilizing immunity. This distinction is essential when interpreting breakthrough infections. Our findings highlight the capacity of mRNA vaccines to establish lasting immunological memory and suggest that vaccine type can influence the magnitude and durability of the response. Notably, both mRNA vaccination and natural infection result in a comparable expansion of memory B cell subsets, reflecting a consistent pattern of humoral immune response across all studied groups.

Ongoing follow-up will be crucial to fully understand the longevity of vaccine-induced immunity, particularly as new variants continue to emerge.

## Figures and Tables

**Table 1:** Distribution of individuals by age, sex, and time point. Within the BNT162b2 group, 50 participants were excluded from subsequent observation at T2 and 56 at T3; within the mRNA-1273 group, 4 participants were excluded at T2 and 6 at T3; within the convalescent group, 13 participants were excluded at T2 and 8 at T3.

	Group		
	BNT162b2 n = 157	mRNA-1273 n = 18	COVID-19 n = 110
Variable			
age (yr.), mean ± SD	53.0, 19.5 *	41.4 ± 10.9	47.4 ± 11.8
sex, n (%)			
male	48 (30.6)	7 (38.9)	39 (35.5)
female	109 (69.4)	11 (61.1)	71 (64.5)
Time point 0, n (%)	42 (26.8)	6 (33.3)	0 (0.0)
Time point 1, n (%)	128 (81.5)	13 (72.2)	54 (49.1)
Time point 2, n (%)	78 (49.7)	9 (50.0)	41 (37.3)
Time point 3, n (%)	22 (14.0)	3 (16.6)	33 (30.0)

* median, IQR.

## Data Availability

Data are contained within the article.

## Abbreviations

The following abbreviations are used in this manuscript:

SARS-CoV-2	Severe Acute Respiratory Syndrome Coronavirus 2
COVID-19	Coronavirus Disease 2019
RBD	Receptor-Binding Domain
MBCs	Memory B Cells
GC	Germinal Center
IgG	Immunoglobulin G
IgD	Immunoglobulin D
IgM	Immunoglobulin M
ELFA	Enzyme-Linked Fluorescent Assay
ELISpot	Enzyme-Linked Immunospot Assay
ASCs	Antibody-Secreting Cells
PBMCs	Peripheral Blood Mononuclear Cells
IL-2	Interleukin-2
SFU	Spot-Forming Unit
NAb	Neutralizing Antibody
RT-PCR	Reverse-Transcription Polymerase Chain Reaction
ANOVA	Analysis of Variance
IQR	Interquartile Range
SEM	Standard Error of the Mean
NK cells	Natural Killer Cells
mAbs	Monoclonal Antibodies
SPSS	Statistical Package for the Social Sciences
FACS	Fluorescence-Activated Cell Sorting
CD	Cluster of Differentiation
BUV, BV, PE, APC etc.	Fluorochromes used in flow cytometry
